# P40 Cross-platform evaluation of AMR social media posts from a national public health body (2025–26)

**DOI:** 10.1093/jacamr/dlag102.046

**Published:** 2026-06-26

**Authors:** Shannon Guild, Tyler King, Sammi Ferhaoui, Diane Ashiru-Oredope

**Affiliations:** UK Health Security Agency, London, UK; UK Health Security Agency, London, UK; UK Health Security Agency, London, UK; UK Health Security Agency, London, UK; University of Bristol, Bristol, UK

## Abstract

**Background:**

Effective antimicrobial resistance (AMR) communication relies on engaging audiences with timely and credible information. Social media offers opportunities to reach public and professional stakeholders, but performance varies across platforms, themes and content types. Engagement rate (ER) represents how frequently users interacted with a post relative to the number of times it was displayed, providing a comparable measure of content effectiveness.

**Objectives:**

To assess the engagement of AMR social media content across platforms, content types and thematic categories.

**Methods:**

A retrospective analysis was conducted on UKHSA AMR related posts published across five social media platforms between March 2025 and February 2026. Extracted metrics included impressions, engagements, reactions, comments and shares from UKHSA’s analytics dataset up to March 2026. Engagement rate (ER=engagements ÷ impressions) was calculated for posts on platforms providing impression data (Facebook, Instagram, LinkedIn, X) and expressed as a percentage. Mean ER was compared across content types, platforms and themes, with posts with zero impressions treated as missing. Platforms without impression data (YouTube and Bluesky) were analysed using engagement volume.

**Results:**

There were 232 AMR related posts across Facebook (*n*=69), Instagram (*n*=45), LinkedIn (*n*=31), X (*n*=54), YouTube (*n*=20) and Bluesky (*n*=13). Posts were categorized by theme as AMR public campaign related (*n*=152), opportunistic (*n*=31) or wider work (*n*=49), and content type as video (*n*=146), photo (*n*=58), carousel (*n*=16), document (*n*=6), text (*n*=4) or link (*n*=2). Mean ER varied across themes and content type. Video produced the highest ER for both campaign posts (mean ER≈2.2%, *n*=131) and wider work posts (mean ER≈6.5%, *n*=12). For opportunistic posts, a single document post generated the highest ER (95.3%), followed by text (mean ER≈34.0%, *n*=2) and carousels (mean ER≈1.9%, *n*=3). Platform level variation was substantial. LinkedIn consistently produced the highest ER across all themes, with mean ER values of 13.3% (*n*=8) for campaign, 41.7% (*n*=4) for opportunistic and 12.9% (*n*=19) for wider work posts. Instagram performed well for video-based campaign content (mean ER≈2.1%, *n*=18), while Facebook performed well for photo-based opportunistic content (mean ER≈2.2%, *n*=9). X contributed modest but stable engagement (mean ER≈1.6%). YouTube and Bluesky demonstrated low engagement volumes overall but supported niche visibility for specific content formats, such as YouTube video and Bluesky photo or link content.

**Conclusions:**

AMR social media engagement varies notably by content format, platform and thematic category. Video is the most effective format overall, particularly within campaign related and wider work themes, while carousels and selected text or document posts can perform strongly within opportunistic messaging. LinkedIn consistently achieved the highest engagement quality across all themes, reflecting strong resonance among professional audiences, while Facebook provided valuable reach during topical periods of heightened public interest. Engagement rate proved to be a meaningful visibility adjusted metric for comparing performance across networks with differing audience sizes. These findings support the development of more targeted, theme specific and platform appropriate AMR communication strategies and highlight the value of expanding analysis to broader datasets to refine future digital engagement approaches.

